# Design and fabrication of an improved dynamic flow cuvette for ^13^CO_2_ labeling in *Arabidopsis* plants

**DOI:** 10.1186/s13007-022-00873-3

**Published:** 2022-03-27

**Authors:** Sonia E. Evans, Peter Duggan, Matthew E. Bergman, Daniela Cobo-López, Benjamin Davis, Ibadat Bajwa, Michael A. Phillips

**Affiliations:** 1 Department of Cell and Systems Biology, University of Toronto, Toronto, ON M5S 3G5 Canada; 2 Academic Machine Shop, University of Toronto-Mississauga, Mississauga, Canada; 3 Department of Biology, University of Toronto-Mississauga, Mississauga, ON L5L 1C6 Canada

**Keywords:** Isotopic labeling, Instrumentation, Gas exchange, Flux analysis, Carbon assimilation, Whole plant physiology

## Abstract

**Background:**

Stable isotope labeling is a non-invasive, sensitive means of monitoring metabolic flux in plants. The most physiologically meaningful information is obtained from experiments that take advantage of the natural photosynthetic carbon assimilation pathway to introduce a traceable marker with minimal effects on the physiology of the organism. The fundamental substrate in isotopic labeling experiments is ^13^CO_2_, which can reveal the earliest events in carbon assimilation and realistically portray downstream metabolism when administered under conditions suitable for making kinetic inferences. Efforts to improve the accuracy and resolution of whole plant labeling techniques have focused on improvements in environmental control, air flow characteristics, and harvesting methods.

**Results:**

Here we present a dynamic flow cuvette designed for single *Arabidopsis thaliana* labeling experiments. We have also verified its suitability for labeling *Nicotiana benthamiana* and essential oils in *Pelargonium graveolens*. Complete plans for fabrication of this device are included. The design includes three important innovations. First, uniform, circular air flow over the rosette surface is accomplished by a fan and deflector that creates a mini-cyclone effect within the chamber interior. Second, a network of circulating canals connected to a water bath provides temperature control to within ± 0.1 ºC under variable irradiance, humidity, and air flow conditions. When photosynthetically active radiation (PAR) was varied over a range of 1000 μEinsteins m^−2^ s^−1^ with no adjustment to the external temperature control system, the abaxial leaf temperature changed by < 3 ºC/1000 PAR. Third, the device is fully compatible with liquid nitrogen quenching of metabolic activity without perturbation of the light environment. For short labeling experiments (< 10 s), the most critical variable is the half-life (t_1/2_) of the atmosphere within the chamber, which determines the maximum resolution of the labeling system. Using an infrared gas analyzer, we monitored the atmospheric half-life during the transition from ^12^CO_2_ to ^13^CO_2_ air at different flow rates and determined that 3.5 L min^−1^ is the optimal flow rate to initiate labeling (t_1/2_ ~ 5 s). Under these conditions, we observed linear incorporation of ^13^C into triose phosphate with labeling times as short as 5 s.

**Conclusions:**

Advances in our ability to conduct short term labeling experiments are critical to understanding of the rates and control of the earliest steps in plant metabolism. Precise kinetic measurements in whole plants using ^13^CO_2_ inform metabolic models and reveal control points that can be exploited in agricultural or biotechnological contexts. The dynamic labeling cuvette presented here is suitable for studying early events in carbon assimilation and provides high resolution kinetic data for studies of metabolism in intact plants under physiologically realistic scenarios.

**Supplementary Information:**

The online version contains supplementary material available at 10.1186/s13007-022-00873-3.

## Introduction

Administration of ^13^CO_2_ as a metabolic tracer in plant metabolism studies has been employed as an experimental technique for nearly half a century [15, 25, 26]. ^13^C is easily detected by mass spectrometry (MS) and nuclear magnetic resonance (NMR), and kinetic isotope effects of ^13^CO_2_ on plant physiology are small [32], providing a unique insight into carbon fixation rates and pathways of photosynthetic organisms under physiological conditions. In addition, stable isotopes such as ^13^C are safer to handle than their radioactive counterparts, offering more experimental flexibility. They also provide compositional and positional information of metabolites via MS and NMR analysis [1]. These factors have led to the near total replacement of ^14^C with ^13^C as the preferred carbon isotope for metabolic studies, although the former is still widely utilized in carbon partitioning and source-to-sink studies [16]. Supplying label in the form of ^13^CO_2_ remains the most physiologically informative and least invasive substrate for metabolic studies of plants.

Multiple reports describe methodologies for conducting stable isotope labeling with ^13^CO_2_ [1, 14, 24]. A sealed flow cuvette has the added benefit of permitting a user defined chase period following administration of label [18]. An important factor in plant isotopic labeling studies is the chamber or cuvette where illuminated plant tissue is brought into contact with the isotopically enriched air under controlled environmental conditions. Modern dynamic flow cuvettes typically feature electronic sensing and control of leaf surface temperature, air flow, and gas composition. Previous designs for dynamic flow cuvettes have been optimized for specific research applications include continuous real time monitoring of volatile emissions in combination with proton transfer reaction mass spectrometry (PTR-MS) [9, 19, 27]. Although short term labeling of experimental plants is more frequently employed, cuvettes designed for long term growth in the presence of ^13^CO_2_ have also been described for protein turnover studies [7], carbon partitioning between organs in grassland vegetation [22], and uniform labeling of wheat using both ^13^C and ^15^N [6].

The first specialized cuvettes to perform short time-course labeling studies with a resolution of ~ 1 min were described nearly 3 decades ago and were capable of labeling small amounts of leaf tissue in addition to trapping volatiles such as isoprene [8]. Schnitzler and co-workers have developed several specialized labeling cuvettes optimized for trees and monitoring isoprene emissions by PTR-MS [17]. The response time of the system was measured at below 1 min in this experimental configuration.

The need to rapidly quench metabolism to make kinetic inferences of labeled metabolites has led to various experimental innovations, including a liquid nitrogen chilled copper rod to freeze tissue in a labeling cuvette [2]. Leaf clamp strategies offer an experimental alternative to whole plant cuvettes to administer ^13^CO_2_ to individual attached leaves and trap emitted volatiles for analysis by gas chromatography—MS [20] or facilitate rapid liquid nitrogen freeze-clamping and quenching of metabolic activity [12]. Other innovations include flooding of the chamber with liquid nitrogen and rapid quenching of metabolism [13]. Addition of liquid nitrogen directly to the chamber to avoid changes in the light environment is essential to study early events in carbon fixation [21].

Improvements in cuvette design and harvesting methods for kinetic analysis has led to finer resolution of the incorporation of ^13^C into metabolite pools adjacent to the Calvin-Benson cycle, such as the 2*C*-methyl-d-erythritol-4-phosphate pool [4, 11, 33] and its downstream products such as stored essential oils [3]. Higher flow rates and reduction of cuvette internal volumes have reduced the shortest reliable labeling times that can be achieved to well under a minute [30]. Here we describe a cuvette design capable of environmental control, rapid atmospheric switching, and in situ liquid nitrogen flash freezing of individual plants following whole plant labeling experiments ranging from several hours down to ~ 5 s. We include complete plans for fabrication of the cuvette at low cost for high resolution kinetic labeling experiments of individual *Arabidopsis* plants.

## Methods and materials

### Plant growth conditions

*Arabidopsis thaliana* ecotype 0 plants were germinated directly in soil (Promix BX MYCORRHIZAE) following 72 h stratification at 4 ºC and cultivated under short day conditions (8 h light/16 h darkness) at 21 ºC and 150 μEinsteins m^−2^ s^−1^ (or photosynthetically active radiation, PAR) using broad spectrum white light emitting diodes (LEDs) (Cree XP-E). The spectral output of growth chamber LEDs was measured with an LI-180 spectrometer (Licor) and compared to that of LEDs used to illuminate the cuvette during labeling experiments to ensure minimal adaptation time of plants prior to initiating labeling (Additional file 1: Fig. S1). Plants were watered twice weekly with tap water and once weekly with MiracleGro soil fertilizer (NPK 20:20:20) prepared according to the manufacturer’s instructions. Plants were grown for 6–8 weeks before use in labeling experiments. All plants were in the pre-flowering, vegetative growth stage at the time of labeling and harvest. *Nicotiana benthamiana* were germinated from seed and grown under long day conditions (16 h day/8 h night) at 25 ºC with 250 PAR. Fertilizer was applied three times weekly, and plants were labeled at 3 weeks of age with 4–6 true leaves. *Pelargonium graveolens* (isomenthone-rich chemotype) were established from cuttings as previously described [3] and grown under the same conditions as *N. benthamiana*. They were labeled before freshly rooted cuttings exceeded 35 mm in height (~ 4 weeks).

### Fabrication of a dynamic flow cuvette

**Fig. 1:**
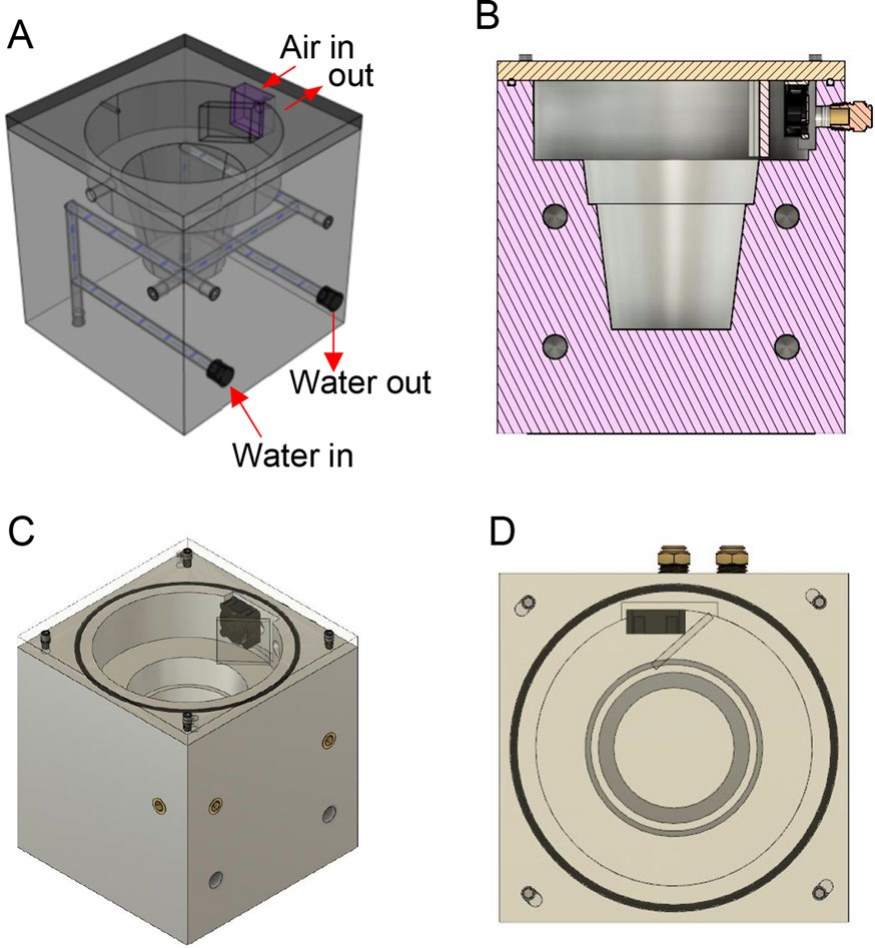
Autocad design of the dynamic flow cuvette. **A** Transparent image showing irrigation canals for temperature control. **B** Side view showing head space and well for pot. **C** Space filling view showing the acrylic lid with O-ring seal, cam-locking screws, deflector, and mixing fan. **D** Top view showing fan and deflector position

**Fig. 2:**
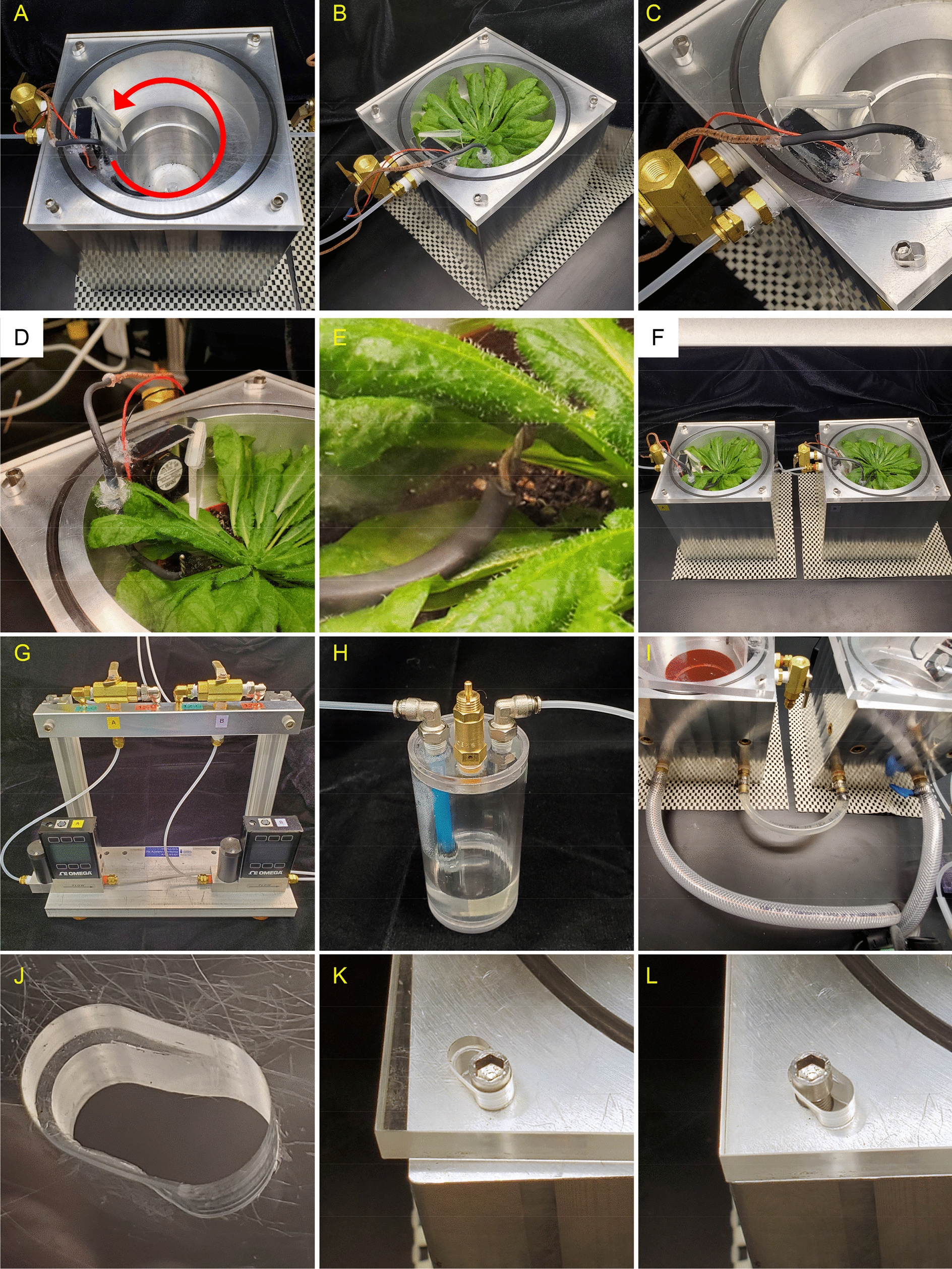
An *Arabidopsis* dynamic flow cuvette for gas exchange measurements and ^13^CO_2_ labeling assays. **A** Top view showing directional air flow (red arrow). **B** The cuvette with *Arabidopsis* plant installed. A ball valve at the exit can vent directly to the atmosphere during liquid nitrogen freezing to protect downstream gas sensors. **C** Close up showing lid-embedded fan, deflector, and thermocouple. **D** Arrangement of leaves around lid components. **E** Thermocouple placement against abaxial leaf surface in sealed cuvette. **F** Gas exchange and labeling assays of a pair of *Arabidopsis* plants in identical cuvettes using a common light source. **G** Gas manifold for tandem labeling cuvettes. A mounted ball valve rapidly switches the gas supply to the cuvette from standard air to ^13^CO_2_-containing air to initiate a labeling experiment. Digital flow controllers maintain constant flow to each cuvette independently. Gas supply to the cuvettes can be configured for tandem or independent operation. **H** A pressurized wash bottle with safety vent adjusts humidity upstream of the gas switching valve. **I** Temperature inside the cuvette is regulated by a series of internal irrigation channels (Fig. 1A) connected to a circulating water bath (not shown). The cuvettes can be linked together as shown to ensure equal temperatures between two experimental plants. **J** Machined camming screw hole for sealing the lid. **K** Lid in loose, open position for rapid removal and liquid nitrogen introduction during flash freezing. **L** Lid locked in sealed position

A single plant, dynamic flow cuvette was designed in Fusion360 (Autodesk) (Fig. 1; for a complete schematic showing all dimensions see Additional file 1: Fig. S2). For fabrication, two identical plant chambers were made from a block of 6061 T6 aluminum. Each cuvette was first machined into a near cube shape measuring 15.24 × 15.24 × 15.875 cm (W × L × H) on a standard knee-milling machine (Ex-Cell-O). Each cube was then transferred to an engine lathe (Misal 815) to drill out an internal, tapered well into the block to accommodate a standard 7.62 cm (3″) plastic pot (A.M.A. Horticulture Inc., Kingsville, ON, USA) which holds a single *Arabidopsis* plant (Fig. 1B). The upper headspace had a total volume of 395 mL to accommodate the rosette. The lathe also introduced a 3 mm groove for an O-ring (14 cm diameter) to seal a clear acrylic lid during use via four cam-locking screws. All remaining machining steps were accomplished on the knee-mill. This included introduction of a 3.0 × 4.2 × 0.4 cm (h × w × d) pocket for a fan, air inlet/exhaust holes, and a lid attachment screw in each corner (Fig. 1C, D). The lid was constructed out of clear acrylic (0.95 cm thickness) cut to fit the outside dimensions of the block. It was machined to accommodate the cam-locking feature and seal the lid during use while also allow rapid removal during harvesting (Fig. 2J–L). A fan and deflector were affixed to the surface, and a thermocouple (National Instruments) was passed through for attachment to the leaf underside. In this way, all cold sensitive electrical components could be removed with the lid as liquid nitrogen is applied for quenching. A 6.4 mm irrigation canal with a total path length of 65 cm was drilled into the block by connecting channels drilled at right angles (Fig. 1A). The entrance and exit holes were threaded with fittings and connected to water hoses, while the others were sealed with brass plugs. Water was circulated through the canals by connecting the hoses to a circulating water bath (VWR) for temperature control inside the cuvette.

### Evaluation of the dynamic flow cuvette in whole plant labeling assays

Labeling assays in the cuvette were carried out on single *Arabidopsis* plants 6–8 weeks of age to calculate atmospheric half-life as a function of flow rate, leaf temperature stability under different light intensities, and the shortest incubation times achievable with the device. Gas was supplied by two tanks connected to the cuvette through a manual valve (Fig. 2G). The first tank contained air with 400 μL L^−1^ CO_2_ (natural isotopic abundance), while the other contained air with ^13^CO_2_ at the same concentration (99% enrichment, Linde Canada). Air flow through each cuvette was controlled by an Omega FMA-2600 digital mass flow controller. Unless otherwise noted, plants were incubated in air at ~ 65% relative humidity, 21 ºC, 150 PAR, and 1.0 L min^−1^ air flow containing 400 μL L^−1^ CO_2_ (standard conditions). Exhaust from the cuvette was directed to a Licor 840a gas analyzer, which registered exchange of the atmosphere when the gas supply was changed, based on the differential sensitivity of the infrared gas analyzer to ^13^CO_2_. Tubing internal diameter and distances were minimized to reduce dead volume between the gas source and cuvette whenever possible.

For a typical gas flow experiment, the wild-type plant was allowed to reach a photosynthetic steady state at a constant flow rate for ~ 30 min in normal air then rapidly switched to the labeling atmosphere as described above for periods ranging from 10 s to 50 min (uncorrected). After switching to the labeling atmosphere, the decay of the ^12^CO_2_ signal was recorded for at least 5 min. The half-life was calculated as the time necessary for the ^12^CO_2_ signal to reach the half-way point between the maximum and minimum signals at each flow rate. Four replicates were performed at each flow rate, and the resulting data points were plotted and fitted to exponential and power functions using Microsoft Excel. For subsequent time course experiments, the individually measured half-life of each labeling experiment was subtracted from the uncorrected labeling time so that the corrected labeling time reflected exposure time starting from a 50/50 mixture of ^12^CO_2_ and ^13^CO_2_-containing atmospheres. The half-life of the atmospheric exchange was calculated individually for each plant labeling experiment.

We next tested the ability of the cuvette to resist temperature changes imposed by changes to incident light intensity. The water bath could be coupled to leaf surface temperature readings to achieve any internal temperature between 4 and 60 ºC at any light intensity, but we temporarily disabled this function to allow an evaluation of the heat sink capacity without water bath temperature adjustment as the light intensity inside the cuvette varied. The heat sink portion of the cuvette base consisted of 9.56 kg aluminum, while the flow rate of water through sealed irrigation canals was approximately 13.5 L min^−1^. The circulating water bath was fixed at 12, 21, or 37 ºC while the light intensity above the cuvette was varied with a potentiometer as described previously [11] from 50 to 1100 PAR at increments of 50 PAR. The leaf surface temperature was allowed to stabilize at each new light setting and recorded while the water bath temperature remained fixed. Single data points were recorded at each light intensity for each of the three temperatures.

To harvest tissue, the rosette was rapidly frozen by flooding the headspace chamber with liquid nitrogen without changing the light environment. Liquid nitrogen was applied directly to leaf tissue as the lid is opened. The expanding nitrogen gas served to prevent unlabeled air from entering the chamber. A circular polytetrafluoroethylene (PTFE) collar was installed around the rosette to facilitate recovery of frozen leaves after freezing. Frozen leaves were transferred to a liquid nitrogen chilled mortar and pestle where they were ground to a fine powder and lyophilized against a vacuum of 0.05 mbar for 72 h. Lyophilized tissue was stored at − 20 ºC until extracted for analysis.

### Analysis of ^13^C label incorporation into metabolites by mass spectrometry

To assess the ability of the device to observe early events in carbon metabolism, we examined the incorporation of ^13^C label into selected central metabolites following time-course labeling of up to 50 min with an emphasis on very short labeling experiments (5 s–4 min). We then prepared polar *Arabidopsis* and *Nicotiana* extracts for liquid chromatography tandem mass spectrometry (LCMS/MS) analysis using HILIC and C_18_ stationary supports as described previously [2, 10] with minor modifications. All steps were carried out at 4 °C. 2-Deoxy-glucose-6-phosphate (2-DGP) was added to each sample as an internal standard prior to extraction. Briefly, lyophilized *Arabidopsis* or *Nicotiana* leaf tissue (10.0 mg) was extracted twice with 250 µL 50% acetonitrile containing 10 mM ammonium acetate (pH 9.0) by vortexing for 30 min. Each extract centrifuged for 15 min at 16,000*g* in a benchtop microcentrifuge. Pooled supernatants were frozen in liquid nitrogen and lyophilized overnight. The residue was dissolved in 100 µL 10 mM ammonium acetate and back extracted with 1 vol chloroform. Following centrifugation, the upper phase was diluted in 1 vol acetonitrile, filtered through a 0.2 µm PTFE syringe filter, and finally transferred to an HPLC vial.

The analytical system consisted of an Agilent 1290 series II UHPLC coupled to a Sciex 4500Qtrap triple quadrupole mass spectrometer operating in multiple reaction monitoring mode with negative electrospray ionization. The following transitions of monoisotopic ions and their isotopologs were monitored with unit resolution: glyceraldehyde-3-phosphate (GAP) and dihydroxyacetone phosphate (DHAP) (*m/z* 169, 170, 171, 172 → 79), 2*C*-methyl-d-erythritol 2,4-cyclodiphosphate (MEcDP) (*m/z* 277, 278, 279, 280, 281, 282 → 79), and isopentenyl diphosphate and dimethylallyl diphosphate (IDP and DMADP) (*m/z* 245, 246, 247, 248, 249, 250 → 79). Labeling calculations were performed as described previously [33].

Ionization settings for MS were as follows: Ion spray voltage − 4500 V, curtain gas 20 psi, collision gas 10 psi, nebulizer gas 60 psi, heating gas 30 psi, and temperature 700 °C. MEcDP, IDP, and DMADP were analyzed on a BEH amide HILIC column (2 mm × 150 mm, 2.5 μm particle size; Waters Corp) as described previously [10]. Analysis of GAP and DHAP was carried out using a Luna C-18(2) column (100 mm × 2.0 mm, 2.5 μm particle size,Phenomenex) to achieve their separation from glycerol-3-phosphate, whose monoisotopic ion is isobaric with their M + 2 ion and thus interferes with calculation of % atom label due to partial co-elution of their chromatographic peaks on the HILIC column. GAP and DHAP could not be separated by either chromatographic support and were analyzed as a single pool. The solvents used were 5 mM tributylamine (TBA) in water with 15 mM acetic acid, pH 4.9 (A) and acetonitrile (B). Separation was achieved at a flowrate of 0.25 mL min^−1^ in with the following gradient: 0–45% B (0–12 min), 45–60% B (12–14 min), 60–90% B (14–15 min), and 0% B (15–20 min). Data processing was performed using SciexOS analysis software (v2.0).

Analysis of labeled essential oils in *Pelargonium graveolens* tissue was carried out by gas chromatography—mass spectrometry (GCMS). Briefly, adapted plants were incubated in an atmosphere containing 400 μL L^−1^ for 3 h, and 100 mg young leaf tissue were immediately steeped in 1 mL ethyl acetate for 8 h at − 20 ºC. Extracts were purified over a short silica column containing MgSO_4_ and analyzed in split mode (1:20) on an Agilent Technologies 7890B gas chromatograph fitted with a 30 m × 0. 25 mm I.D. polyethylene glycol capillary column (Innowax). Data were acquired in scan mode (*m/z* 50–225), and (–)-isomenthone labeling was determined by integrating extracted ion chromatograms *m/z* 154–164 corresponding to its monoisotopic ion and variously labeled isotopologs. All other analytical conditions were as described previously [3]. Statistical significance for ^13^C enrichment was tested with a two-tailed Student’s t-test using Microsoft Excel.

## Results and discussion

### A novel dynamic flow cuvette design facilitates detailed examination of plant metabolism

We present a design for a temperature controlled, dynamic flow cuvette for gas exchange measurements on a single *Arabidopsis* plant that was capable of both rapid switching to a ^13^CO_2_ containing atmosphere as well as in situ flash freezing (Figs. 1, 2). The latter criterion is necessary to avoid perturbation of the light environment during harvesting, which would alter the concentrations of rapidly turned over intermediates within the Calvin-Benson cycle and downstream pathways closely connected to it [13]. The design consists of a machined aluminum block (Fig. 2A, B), an acrylic lid (Fig. 2C), and a network of water circulation canals for temperature control (Fig. 2I). Water circulation through the canals regulates the temperature but does not contact the plant. The inlet and outlet of the irrigation channels are connected to a standard laboratory circulating water bath that can be adjusted manually or linked to a control system. Humidity is controlled by passing air directly from the tank to a custom wash bottle with an integrated pressure release valve (Fig. 2H) that can be chilled or warmed according to the desired relative humidity.

Air flow is accomplished by connection to pressurized air tanks calibrated to 400 μL L^−1^ CO_2_ or ^13^CO_2_ (standard and labeling air, respectively). While the use of two premixed air tanks lacks some experimental flexibility, such as the ability to change CO_2_ or O_2_ concentrations, the benefits are reduced cost, simplified operation, and improved reproducibility between labeling assays over time. Gas exchange monitoring in standard air indicates that rosette stage *Arabidopsis* plants typically reached a photosynthetic steady state within 30 min of placement in the cuvette under standard conditions (see “Methods and materials”). Incubation in a labeling atmosphere can be carried out under constant environmental conditions in this cuvette for a period ranging from a few seconds to days. The rapid atmospheric switching design allows either a constant pulse of labeled air until liquid nitrogen freezing or a chase period of any length by reintroducing the original atmosphere for an arbitrary time. Our performance evaluation focused on examining the earliest events of carbon metabolism and therefore generally consisted of pure labeling without a chase period prior to freezing. The leaf surface temperature is monitored directly by a thermocouple in constant contact with the abaxial leaf surface. The deflector directs the incoming air around the cuvette in a circular motion, creating a mini-cyclone effect within the headspace that evenly exposes the rosette surface to a constant supply of fresh air. The thermocouple, air circulating fan, and deflector are attached to the acrylic lid. Liquid nitrogen is introduced as the lid is removed, preventing damage to the electrical components. For comparative studies, two identical cuvettes could be operated simultaneously (Fig. 2F). Using this dual cuvette system, a single user can label and harvest up to 10 plants in a single day while avoiding the first and last two hours of the day to minimize diurnal effects. Our system accommodates large *Arabidopsis* rosettes as well as any other similarly sized plant, providing up to 300 mg DW of ground, lyophilized tissue per plant that can be repeatedly sampled for downstream MS or NMR analyses.

### Performance evaluation of the cuvette

**Fig. 3:**
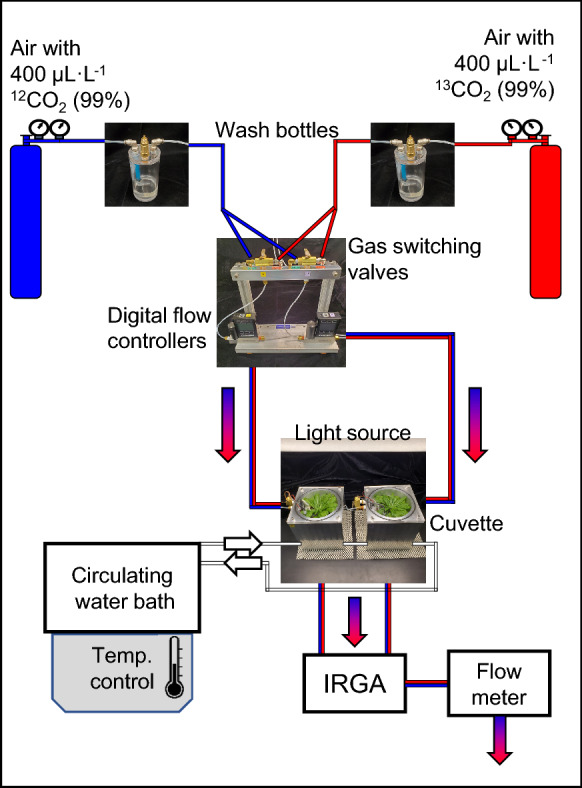
Schematic overview of a dynamic flow cuvette and ^13^CO_2_ whole plant labeling system. Not shown: a data system logs leaf temperature and gas exchange parameters. The infrared gas analyzer (IRGA), water bath, and flow controllers are third party appliances. The remaining components were designed and fabricated for this study. The plumbing configuration shown supports fully independent operation of the two cuvettes. Red and blue gradient arrows signify air flow that changes from standard air to labeled air during a labeling experiment

We first tested the integrity and seal of the fully assembled system under standard air flow conditions (21 ºC, 1.0 L min^−1^; see Fig. 3 for a schematic of a typical experimental setup). The tanks, wash bottles, flow controllers, cuvettes, and gas sensors were connected with 3.2 mm (1/8″) I.D. tubing and brass push connect fittings to reduce dead volumes. Since these connections represented potential sources of leakage, we monitored the total flow into and out of the cuvette. With the upstream flow controller set to 1.0 L min^−1^, we measured a flow of ≥ 0.95 L min^−1^ exiting the gas sensor, indicating that no more than 5% of the air flow was lost through the headspace lid and tubing connections. The cuvette was empty during this measurement to avoid changes to the air volume due to transpiration. The lid forms an effective seal against the wetted O-ring through the action of four camming screws which push the lid downward when locked into place (Fig. 2J–L). The lid was identified as a more significant source of leakage than tubing or push connectors, but the loss of total air flow was minimal when the cuvette was properly sealed.

**Fig. 4:**
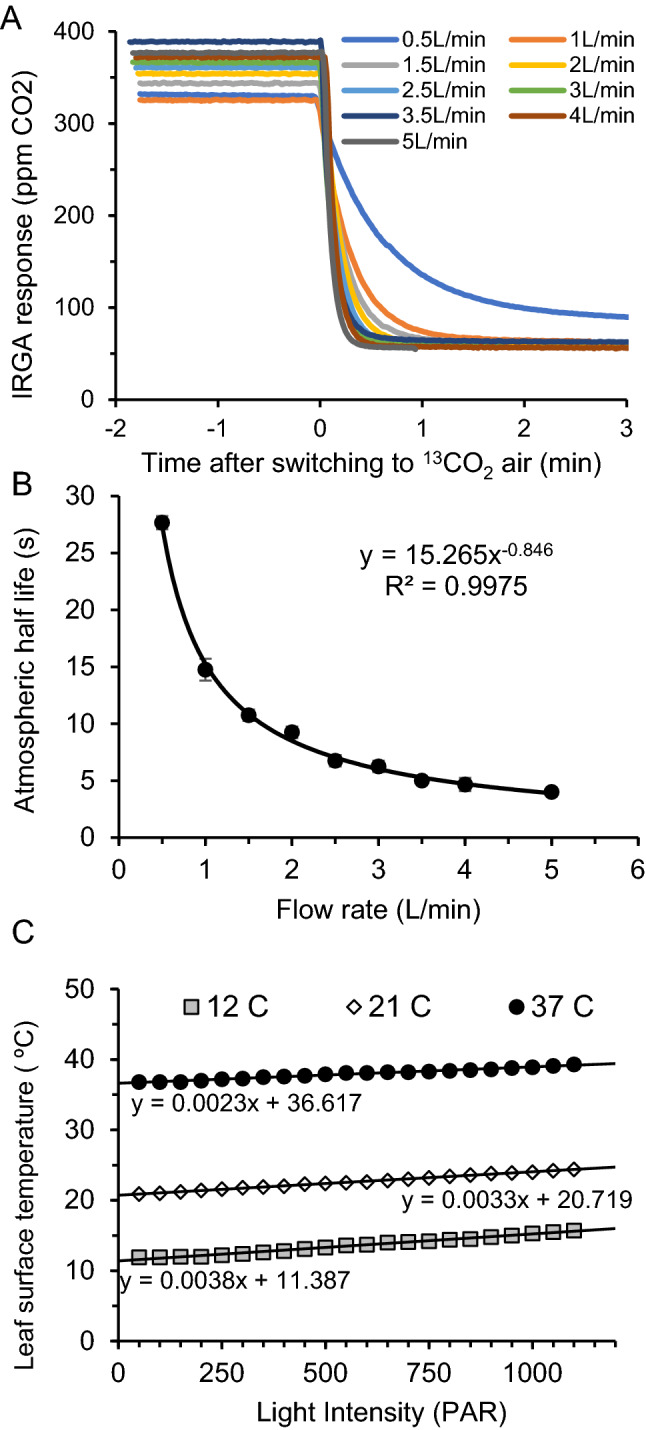
Performance evaluation of the dynamic flow cuvette. **A** The replacement time of the atmosphere in the cuvette head space was measured at different flow rates by making a step change to a ^13^CO_2_ containing air and observing the decay of the IRGA signal. **B** Calculated half-life of air in the cuvette at different flow rates. The data were fitted to a power function in Excel. All air flow experiments were performed with a plant inside the cuvette. **C** Resistance of the cuvette to temperature changes at increasing light intensity. The water bath set point was fixed as light intensity varied to assess the ability of the block to maintain temperature across a light intensity gradient. Temperatures reflect the abaxial leaf surface inside the cuvette using a thermocouple

Atmospheric half-life calculations were derived from ^12^CO_2_ decay curves upon switching to a ^13^CO_2_ containing atmosphere (Fig. 4A). Although the total CO_2_ concentration was constant during a step change to isotopically labeled air, the decreased sensitivity of the IRGA for ^13^CO_2_ provided a means to monitor the replacement rate of air in the cuvette. Labeling times were also corrected from these CO_2_ decay curves on an individual basis, which were especially important for short labeling experiments. This correction consisted of measuring the time needed to reach a 50:50 mixture of the two atmospheres from the moment the gas source is changed and subtracting this value from the total labeling time. We measured the decay of the ^12^CO_2_ signal at flow rates ranging from 0.5 to 5.0 L min^−1^ (Fig. 4B; n = 4 plants per flow rate). We observed an exponential decrease in half-life as air flow increased, reaching a minimum value of ~ 4 s at the highest flow rate employed. This was only slightly longer than the predicted half-life for a cuvette with headspace volume of 395 mL at this flow rate (3.2 s), given by τ = ln2(V/F), where V and F represent the volume (L) and flowrate in L s^−1^. This small increase was likely the result of wind resistance imposed by the irregular leaf surface of the rosette within the chamber. These results suggested that further increases in flow rate beyond 5.0 L min^−1^ would not significantly shorten the atmospheric exchange rate. Indeed, based on these results, we selected 3.5 L min^−1^ as the optimal flow rate in this system that balances an acceptably short atmospheric replacement time (t_1/2_ ~ 5.3 s) with conservation of the comparatively expensive labeled air mixture. In practice, we obtained the best results by programming the flow controller to temporarily increase the flow rate from 1.0 L min^−1^ to 3.5 L min^−1^ shortly before switching to the labeling atmosphere and maintaining this increased flow rate until the previous atmosphere had been essentially replaced. After several minutes, the flow rate was returned to the initial flow rate (1.0 L min^−1^) for the remainder of the labeling experiment.

We next evaluated the capacity of the cuvette body to resist changes in internal temperature when the light intensity varied. Any combination of light intensity and leaf surface temperature could be achieved by adjusting the set points accordingly. However, when we fixed the water bath at a constant temperature of 12, 21, or 37 ºC and increased the light intensity from 50 to 1100 PAR, we observed only very modest changes to the leaf surface temperature (Fig. 3C). Based on linear regression analysis of the fitted data, we observed a change of 3.8 ºC, 3.3 ºC, and 2.3 ºC for each 1000 PAR increase in light intensity when the water bath temperature was held constant at 12, 21, or 37 ºC, respectively. This indicated that the water circulation system has sufficient heat absorbing capacity to absorb a large increase in light intensity with only a small change in leaf temperature for experiments conducted under chilling (12 ºC), standard (21 ºC), or heat stress conditions (37 ºC). The temperature the system could maintain at the leaf surface by modifying the water bath temperature ranged from 4.4 ºC to ~ 66 ºC under ambient laboratory conditions. Thus, the cuvette provided control of temperature and light intensity independently with broad set points that covered a wide range of physiologically plausible scenarios.

### Linearity of label incorporation into primary metabolites following short labeling experiments

An important consideration for performing whole plant labeling experiments is the experimental resolution for making kinetic inferences, as determined by the shortest labeling experiment that can be accomplished by a cuvette system [13]. To study early events in carbon assimilation, it is advantageous to administer labeled CO_2_ for as short a time as possible before quenching metabolism by freezing. We therefore carried out a time-course labeling series that included short labeling experiments (< 1 min) to determine the minimum labeling time that provided reproducible results in this cuvette. Assuming equal mixing throughout the chamber, after 1 half-life (~ 5 s), the CO_2_ in the headspace atmosphere should reach an isotopic enrichment of approximately 50%. Labeling experiments between 5 s and 50 min were performed, and we then examined the % atom labeling into a variety of phosphorylated metabolites from central metabolism and plotted these against the labeling time.

**Fig. 5:**
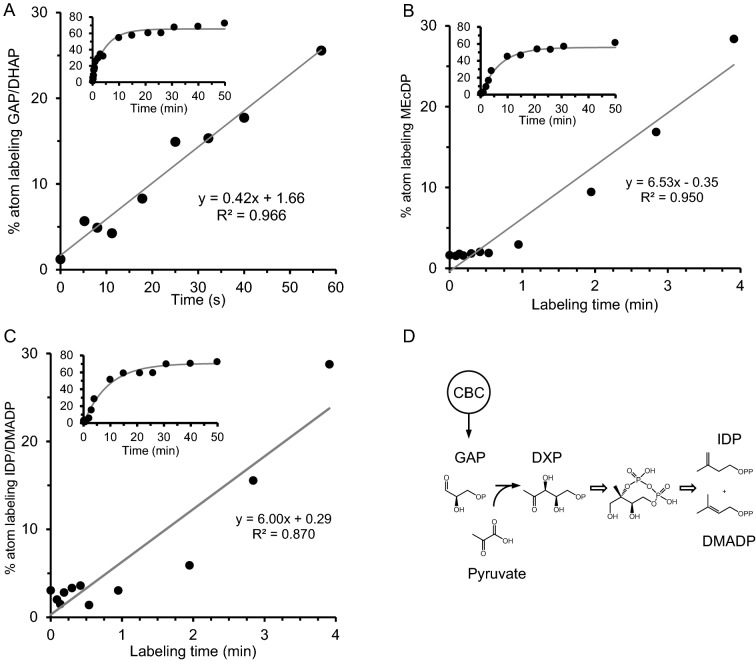
Incorporation of ^13^C into intermediates of primary metabolism following ^13^CO_2_ exposure, as determined by LCMS/MS analysis of labeled plant tissue. Labeling of triose phosphate (**A**), 2*C*-methyl-d-erythritol-2,4-cyclodiposphate (MEcDP, **B**), and isopentenyl and dimethylallyl diphosphate (IDP + DMADP, **C**) are shown. Each time point represents a separate experiment where an individual *Arabidopsis* plant was first adapted, then labeled with air containing 400 μL L^−1 13^CO_2_, and finally flash frozen. Labeling assays ranging from 5 s to 50 min (n = 19 plants) were performed to examine the linearity of short labeling assays and the initial slope (rate) of label incorporation by fitting the earliest time points to a linear regression. The embedded graphs inside each panel show the full time-course fitted to an exponential rise to maximum. **D** Overview of the metabolic sequence from the Calvin-Benson cycle (CBC) to IDP and DMADP in the chloroplast indicates their expected order of labeling. Hollow arrows indicate multiple steps and OP signifies a phosphate group and OPP a pyrophosphate. GAP, d-glyceraldehyde-3-phosphate; DXP, 1-deoxy-d-xylulose-5-phosphate

Figure 5 shows incorporation of ^13^C into triose phosphate and three downstream intermediates of the 2C-methyl-d-erythritol-4-posphate (MEP) pathway, methyl-d-erythritol-2,4-cyclodiphosphate and the isopentenyldiphosphate and dimethylallyl diphosphate pool (IDP/DMADP) in time-course labeled *Arabidopsis* plants. IDP and DMADP cannot easily be resolved chromatographically and were examined collectively here. Figure 5 shows total label incorporation into the metabolite pool, and relative abundances of individual isotopologs are displayed in Additional file 1: Fig. S3. ^13^C enrichment in triose phosphate pools of unlabeled control plants was ~ 1.2%, consistent with expected natural abundance values. Although the analysis of highly polar analytes is generally superior on a HILIC stationary support, we relied on a C_18_ reverse phase column with TBA ion pairing reagent for triose phosphate isotopolog analysis (Additional file 1: Fig. S4). This overcame the problem of co-elution with glycerol-3-phosphate on a HILIC column, whose negative mode monoisotopic ion (*m/z* 171) coincides with the M + 2 isotopolog of triose phosphate. Incorporation of ^13^C into triose phosphate of labeled plants was detectable at the earliest time point (5 s) and increased in a linear fashion over the first minute with a slope of 0.42%/s (Fig. 5A). This is generally consistent with previous reports of triose phosphate labeling [30] and modeled estimates of flux through the plastidic GAP dehydrogenase step [21] except that we do not observe any lag time to triose phosphate labeling. The linearity of ^13^C incorporation into triose phosphate in the first minute (R^2^ = 96.6%) provides strong support for the effectiveness of using the atmospheric half-life as a reference point for labeling experiments. The full triose phosphate time-course (Fig. 5A, inset) displays a hyperbolic shape and was fitted to an exponential rise to maximum with a plateau labeling value of ~ 60%, a rate constant of 0.196, and a calculated half-life of 2.47 min. This turnover rate is slower than previously reported estimates [29] but also includes triose phosphate in the cytosol. To our knowledge, this represents the finest resolution of triose phosphate labeling obtained to date.

Total ^13^C-enrichment of MEP pathway intermediates MEcDP and IDP/DMADP showed a lag time of 30–45 s and 1–2 min, respectively, before label was detected above that of unlabeled controls (Fig. 5B, C). This is consistent with their position downstream from the Calvin-Benson cycle, and this lag likely reflects the time necessary to undergo conversion through the initial steps of the MEP pathway [23]. The plateau labeling levels of all three metabolites approach ~ 60% over the 50 min time-course. The rapid atmospheric switching and harvesting enabled by the present cuvette design substantially improves resolution of the early steps of labeling of the MEP pathway [33].

### ^*13*^*CO*_*2*_* labeling of other model and non-model plants species*

**Fig. 6:**
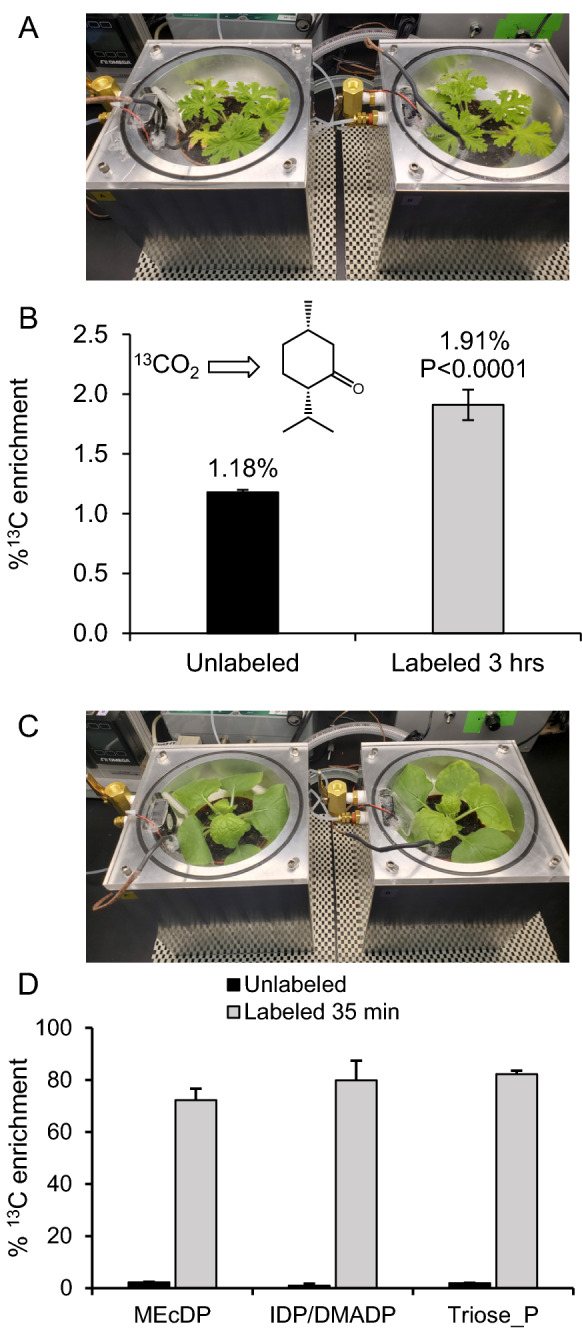
^13^CO_2_ labeling of additional plant species. **A** Rose-scented geranium (*Pelargonium graveolens*) in a tandem cuvette labeling experiment. **B** Labeling of the monoterpene (–)-isomenthone in glandular trichomes of *P. graveolens* after 3 h in 400 μL L^−1 13^CO_2_ at 250 PAR light intensity. **C** *Nicotiana benthamiana* labeled in the same cuvette for 35 min. **D** ^13^C enrichment of primary metabolites in *N. benthamiana*, including triose phosphate, 2*C*-methyl-d-erythritol-2,4-cyclodiposphate (MEcDP), isopentenyl diphosphate (IDP), and dimethylallyl diphosphate (DMADP). Error bars signify standard deviation (n = 4). *Triose_p* triose phosphate

The cuvette was designed to take advantage of the discoid growth habit of *Arabidopsis thaliana*, which not only features rich genetic resources and an annotated genome but also presents an aerodynamically favorable shape for gas flow studies. However, we further examined the utility of this device with other plant species. We chose two plant species representing two extremes in plant metabolism: the model system *Nicotiana benthamiana*, which grows rapidly and is highly amenable to transient gene expression, and the non-model system *Pelargonium graveolens*, which slowly incorporates ^13^C into essential oils in glandular trichomes, especially the *p*-menthane monoterpene (–)-isomenthone [5]. Due to the continuous accumulation of essential oils in glandular trichomes, a high background of unlabeled material is present, and observed labeling above background levels is typically on the order of 1–3%. As shown in Fig. 6, *P. graveolens* plants incubated in the cuvette with ^13^CO_2_ containing air for 3 h showed a statistically significant enrichment of ^13^C into its principal monoterpene (–)-isomenthone (1.91%) above background natural isotopic abundance (1.18%,p < 0.0001), which verified that the cuvette is suitable for labeling of slowly synthesized secondary metabolites in this non-model species. This single time point experiment is consistent with previous time-course analyses of essential oil labeling in *P. graveolens* performed in large format cuvettes designed for adult plants [3]. However, in the present case, this cuvette design improves the resolution of labeling experiments that can be performed and reduces the half-life of the cuvette atmosphere from ~ 10 min to ~ 5 s.

When we labeled *N. benthamiana* in the cuvette, we observed a similar and rapid incorporation of ^13^C into primary metabolites seen in Arabidopsis (Fig. 6C, D). After 35 min of labeling, the enrichment of ^13^C in the triose phosphate pool had reached 82.3%, and a similar level of enrichment was observed for MEcDP and IDP/DMADP (72.2% and 79.9%, respectively.) These results confirmed that this cuvette design could, in principle, study other plant species besides *Arabidopsis* and that both primary and secondary metabolism were amenable to investigation with this device.

## Conclusions

The use of ^13^CO_2_ as a universal tracer is now the established norm in metabolic flux experiments [21, 30, 31]. However, a rigorous evaluation of the flow characteristics and temperature stability of the cuvette is not frequently reported to establish, for instance, the limit to which kinetic inferences may be drawn from time course analysis of plants. The approach described here, wherein the half-way point during atmospheric exchange is utilized as a common starting point in labeling experiments, improves the linearity of fractional labeling measurements, especially for labeling experiments under 1 min. The dynamic flow cuvette design presented here for single *Arabidopsis thaliana* labeling experiments is provided with complete plans for fabrication and can accommodate other plant species with a similar growth habit. It can be constructed by lathing a solid aluminum block to fit the contours of a single pot as described here or, alternatively, by 3D printing to reduce costs. The design includes three important innovations. First, uniform, circular air flow over the rosette surface is accomplished by a fan and deflector that creates a mini-cyclone effect within the chamber interior. Second, a network of circulating canals connected to a water bath provides temperature control to within ± 0.1 ºC under variable irradiance, humidity, and air flow conditions. Third, the device is fully compatible with liquid nitrogen quenching of metabolic activity without perturbation of the light environment. Thus, it is suitable for studying early events in carbon assimilation and photosynthetic metabolism. We determined that labeling experiments shorter than the atmospheric half-life are impractical, and for this reason, the most critical performance parameter of a labeling system is the half-life of the atmosphere within the chamber, which determines the maximum resolution of the labeling system.

Using this custom device, additional efforts to refine short labeling experiments to illuminate the earliest events of plant metabolism can be addressed. This includes plants genetically manipulated in carbon fixation enzymes as well as sugar phosphate transporters. Indeed, incomplete labeling of Calvin-Benson cycle intermediates has been attributed to reimport of carbon from the pentose phosphate pathway to replenish carbon pools in the chloroplast [28]. This device, in combination with the relevant *Arabidopsis* mutants or agroinfiltrated *N. benthamiana*, holds significant potential for investigating carbon fixation, transport of sugar phosphates, photorespiration, and other processes within primary metabolism. The environmental control features allow carbon fixation under light, chill or heat stress to be investigated using this device. Finally, based on the isotopic labeling of whole *P. graveolens* plants, the comparatively slow metabolism of essential oil biosynthesis and storage in glandular trichomes of non-model plant species can similarly be analyzed in detail, enabling broad inquiries of myriad specialized metabolites with roles in medicine, biotechnology, and agriculture.

## Supplementary Information

**Additional file 1**: **Fig. S1**. Spectral output of light emitting diodes (LEDs) used in growth chamber and labeling experiments. The measured light spectrum of natural sunlight is provided for comparison. All spectra were measured with a Licor LI-180 radio spectrometer. PPFD, photosynthetically active photon flux density (μEinsteins m^-2^ s^-1^). **Fig. S2**. Schematic of cuvette design created in Fusion360 (Autodesk). All dimensions are shown in mm. **Fig. S3**. Relative distribution of ^13^C labeled isotopologs of the central metabolic intermediates described in Figure 5 during a whole plant time-course labeling series. A, Relative isotopolog abundance of triose phosphate. B, Relative isotopolog abundance of MEcDP. C, Relative isotopolog abundance IDP & DMADP. See “Methods and materials” for additional details on the acquisition of metabolite labeling data. **Fig. S4**. Representative LCMS/MS and GCMS chromatograms of ^13^C labeled plant metabolites analyzed in this study. A-D were acquired by LCMS/MS in multiple reaction monitoring mode and E by GCMS. A, Separation of triose-phosphate and glycerol 3-phosphate standards (black line = *m/z* 169 → 79; orange line = *m/z* 171 → 79). B, Triose-phosphate in labeled Arabidopsis extracts showing individual isotopologs (*m/z 169 – 172 → 79*) after 30 min labeling and their separation from glycerol-3-phosphate *(m/z* 171 *→ 79).* A and B were resolved on a Luna C-18(2) column (100 mm × 2.0 mm, 2.5 mm particle size; Phenomenex) (see “Methods and materials”). C, Analysis of 2*C*-methyl-D-erythritol-2,4-cyclodiposphate (MEcDP, *m/z 277 → 79*). D, Isopentenyl and dimethylallyl diphosphate (IDP+DMADP, *m/z* 245 *→ 79*). C and D were separated on a HILIC column. E, GCMS analysis of (-)-isomenthone in a *Pelargonium graveolens* leaf surface extract.

## Data Availability

All data are available as figures and supporting figures.
