# Supplementary material for: Design and fabrication of an improved dynamic flow cuvette for 13CO2 labeling in Arabidopsis plants
Source: Plant Methods. 2022 Mar 27;18:40. doi: 10.1186/s13007-022-00873-3 (PMC8958768; doi:10.1186/s13007-022-00873-3)
Supplement: Supplementary file 1 — Additional file 1: Fig. S1. Spectral output of light emitting diodes (LEDs) used in growth chamber and labeling experiments. The measured light spectrum of natural sunlight is provided for comparison. All spectra were measured with a Licor LI-180 radio spectrometer. PPFD, photosynthetically active photon flux density (μEinsteins m-2 s-1). Fig. S2. Schematic of cuvette design created in Fusion360 (Autodesk). All dimensions are shown in mm. Fig. S3. Relative distribution of 13C labeled isotopologs of the central metabolic intermediates described in Figure 5 during a whole plant time-course labeling series. A, Relative isotopolog abundance of triose phosphate. B, Relative isotopolog abundance of MEcDP. C, Relative isotopolog abundance IDP & DMADP. See “Methods and materials” for additional details on the acquisition of metabolite labeling data. Fig. S4. Representative LCMS/MS and GCMS chromatograms of 13C labeled plant metabolites analyzed in this study. A-D were acquired by LCMS/MS in multiple reaction monitoring mode and E by GCMS. A, Separation of triose-phosphate and glycerol 3-phosphate standards (black line = m/z 169 → 79; orange line = m/z 171 → 79). B, Triose-phosphate in labeled Arabidopsis extracts showing individual isotopologs (m/z 169 – 172 → 79) after 30 min labeling and their separation from glycerol-3-phosphate (m/z 171 → 79). A and B were resolved on a Luna C-18(2) column (100 mm × 2.0 mm, 2.5 mm particle size; Phenomenex) (see “Methods and materials”). C, Analysis of 2C-methyl-D-erythritol-2,4-cyclodiposphate (MEcDP, m/z 277 → 79). D, Isopentenyl and dimethylallyl diphosphate (IDP+DMADP, m/z 245 → 79). C and D were separated on a HILIC column. E, GCMS analysis of (-)-isomenthone in a Pelargonium graveolens leaf surface extract. [file 13007_2022_873_MOESM1_ESM.pdf]

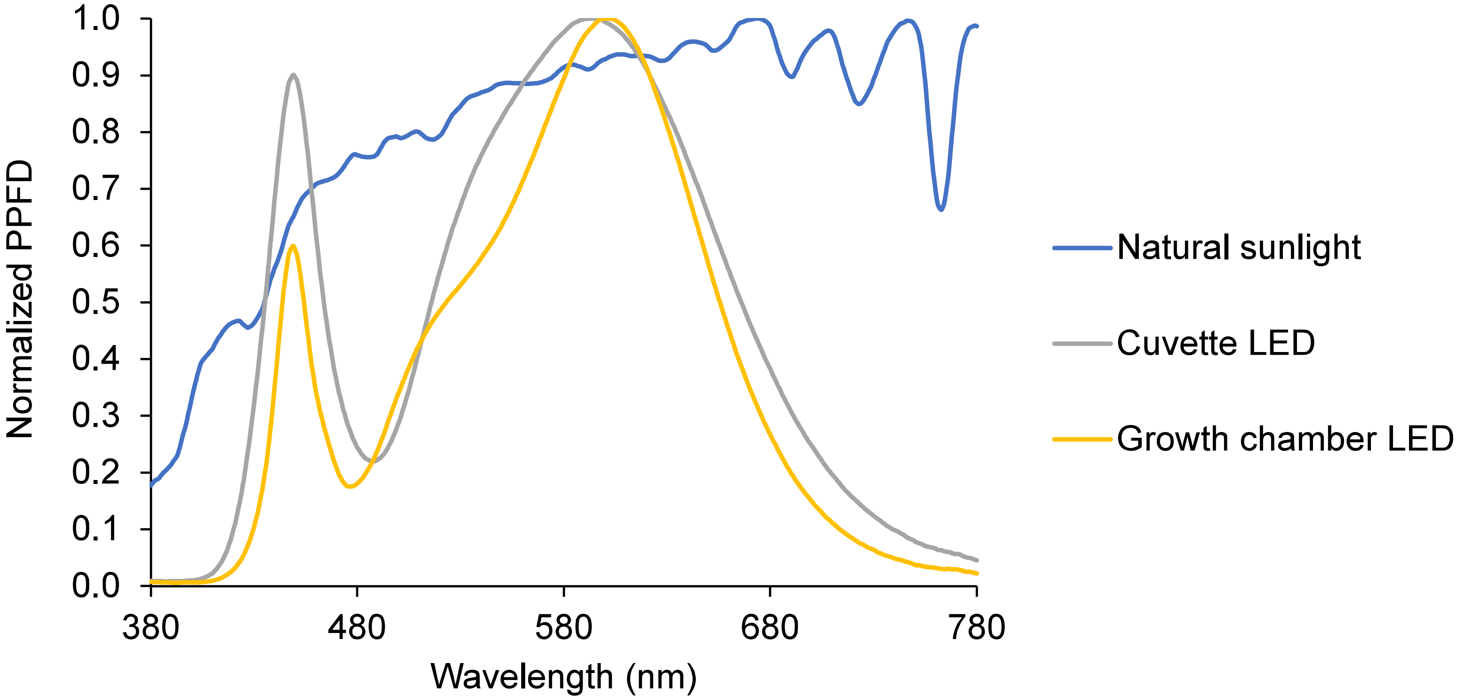

Supporting figure S1

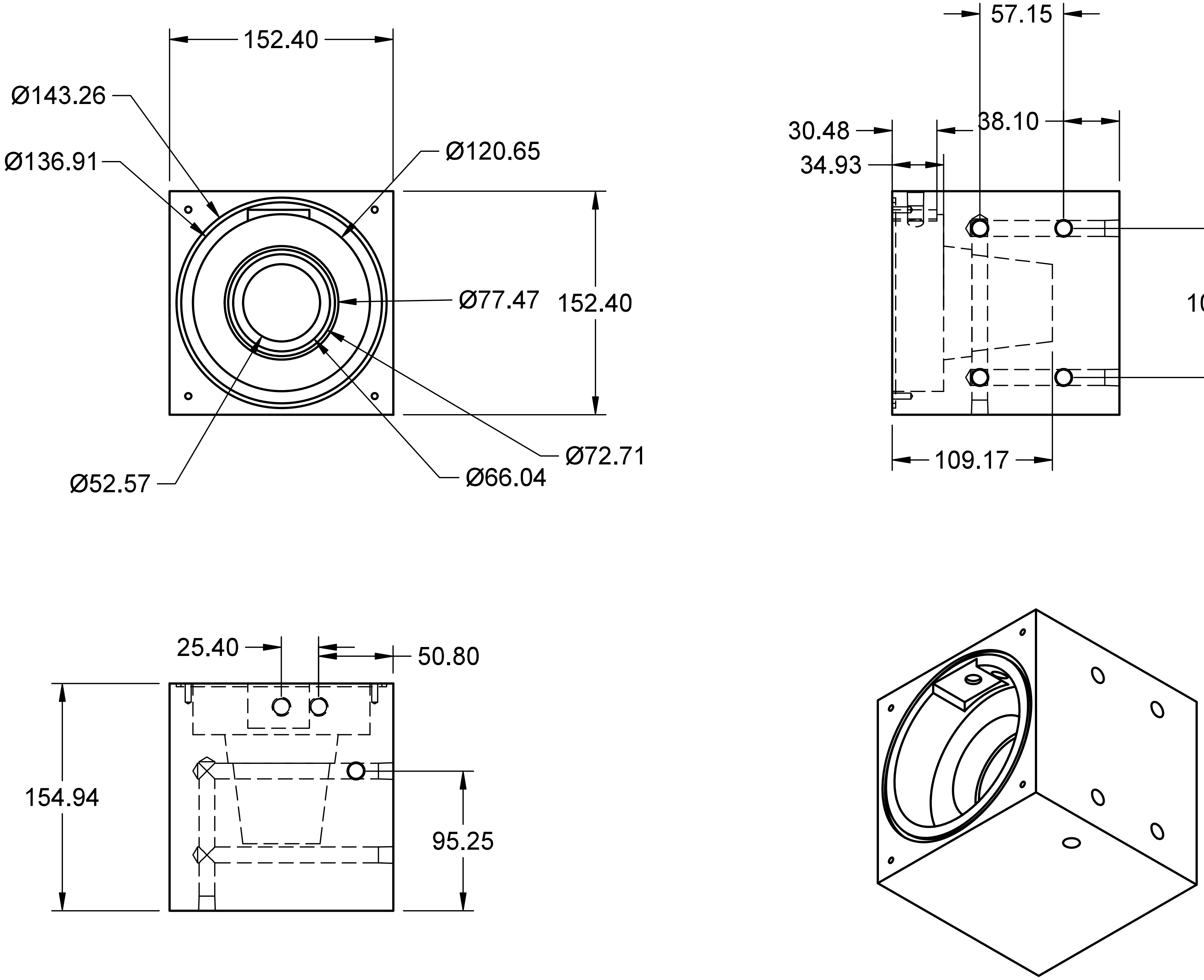

Supporting figure S2. Schematic of cuvette design created in Fusion360 (Autodesk). All dimensions are shown in mm.

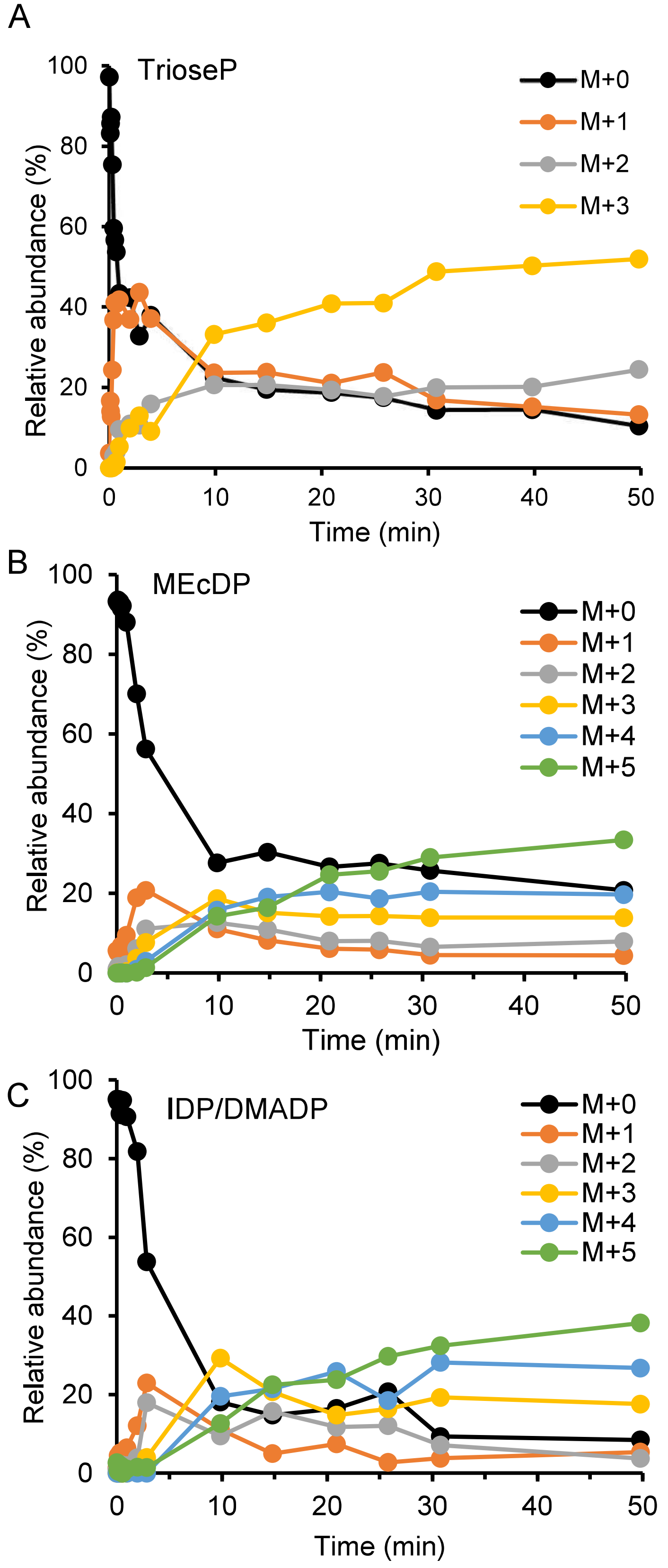

Supporting figure S3.

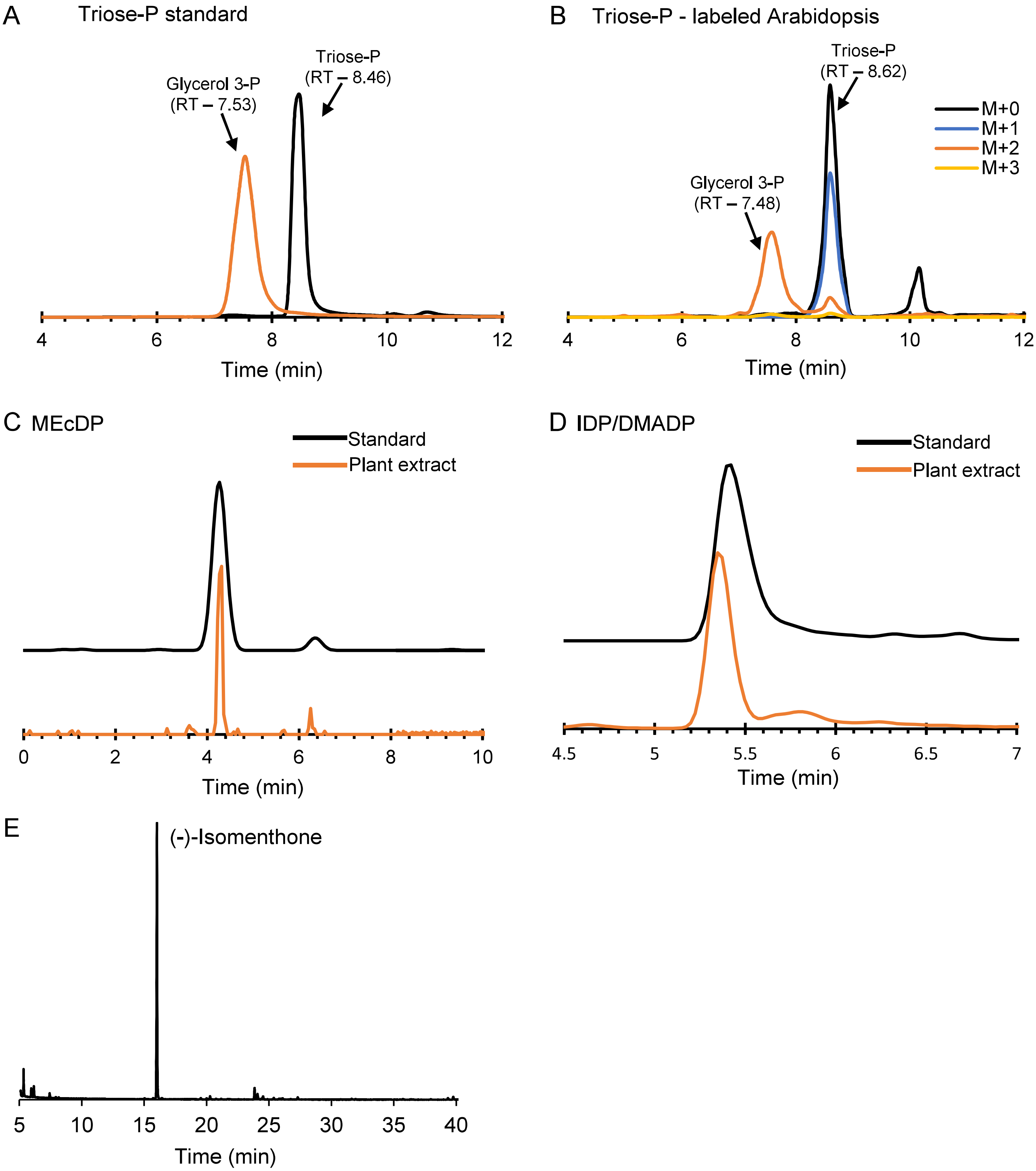

Supporting figure S4
